# Preparation and Properties of Melamine Urea-Formaldehyde Microcapsules for Self-Healing of Cementitious Materials

**DOI:** 10.3390/ma9030152

**Published:** 2016-03-03

**Authors:** Wenting Li, Xujing Zhu, Nan Zhao, Zhengwu Jiang

**Affiliations:** Key Laboratory of Advanced Civil Engineering Materials of Ministry of Education, Tongji University, Shanghai 201804, China; lwt@tongji.edu.cn (W.L.); cumtzhuxujing@163.com (X.Z.); xsy1331@163.com (N.Z.)

**Keywords:** microencapsulation, self-healing, cementitious, urea-formaldehyde

## Abstract

Self-healing microcapsules were synthesized by *in situ* polymerization with a melamine urea-formaldehyde resin shell and an epoxy resin adhesive. The effects of the key factors, *i.e*., core–wall ratio, reaction temperature, pH and stirring rate, were investigated by characterizing microcapsule morphology, shell thickness, particle size distribution, mechanical properties and chemical nature. Microcapsule healing mechanisms in cement paste were evaluated based on recovery strength and healing microstructure. The results showed that the encapsulation ability, the elasticity modulus and hardness of the capsule increased with an increase of the proportion of shell material. Increased polymerization temperatures were beneficial to the higher degree of shell condensation polymerization, higher resin particles deposition on microcapsule surfaces and enhanced mechanical properties. For relatively low pH values, the less porous three-dimensional structure led to the increased elastic modulus of shell and the more stable chemical structure. Optimized microcapsules were produced at a temperature of 60 °C, a core-wall ratio of 1:1, at pH 2~3 and at a stirring rate of 300~400 r/min. The best strength restoration was observed in the cement paste pre-damaged by 30% f_max_ and incorporating 4 wt % of capsules.

## 1. Introduction

Concrete is one of the most widely used construction materials. It is susceptible to crack formation when exposed to certain environmental conditions and external loads. The cracks undoubtedly endanger the performance and potential service life of concrete structures in terms of their mechanical and/or transport properties [1,2,3,4]. Accordingly, immediate cracks repair in concrete is a property that is strived for.

A bio-inspired self-healing capability, like natural healing of wounds or cuts in living species, has become the focus of increasing attention because it potentially enables restoration of the structural integrity of materials [5,6,7,8]. Similar ideas have been incorporated to endow concrete materials a built-in self-healing ability since it was originally proposed for cement matrix by Dry [9,10,11,12,13,14,15,16]. A variety of approaches have been developed based on experimental explorations, utilizing bacteria spores [17,18,19,20,21,22,23], mineral admixtures [24,25,26,27,28,29,30,31,32], tabulated capsules or microencapsulation, *etc*. [33,34,35,36]. 

Encapsulation techniques have attracted increasing interest in self-healing cementitious materials due to that transfer of stress between failed crack faces can be rebuilt when polymers are used as the adhesive that can provide fast recovery of strength compared to other approaches, in addition to the improved transport properties. Potential healing occurs upon rupture of the capsules by propagating cracks and release of both components which then contact and polymerize each other [5]. Microencapsulation of various combinations of healing agents and shell wall has been conducted to achieve desired healing efficiency. Epoxy resin and urea-formaldehyde are one of the most commonly used matches in microencapsulation since epoxy has been normally used for repair of concrete. Behzadnasab [37] synthesized UF microcapsules filled with linseed oil in various core-wall ratios and different mixing speeds. The healing efficiency was evaluated according to the mechanical response of epoxy-based coatings containing various amounts of microcapsules. Only the morphology and size of the microcapsules were characterized without its mechanical properties included. Blaiszik *et al*. [38] studied an *in situ* encapsulation method for reactive epoxy resins core with different diluents surrounded by a urea-formaldehyde (UF) shell. The capsules are demonstrated to satisfy the requirements for use in self-healing of epoxy-based composites including processing survivability, thermal stability, and efficient *in situ* rupture for delivery of healing agent. The influence of the processing parameters on the microcapsules is unknown as well as the mechanical properties of the shell. Jin *et al*. [39,40] synthesized hollow PUF polymeric microcapsules containing epoxy-amine and demonstrated the excellent healing performance of epoxy matrix incorporating the capsules. Tripathi *et al*. [41] compared the influence of melamine-formaldehyde (MF) and urea-formaldehyde (UF) as the shell material on the mechanical response of epoxy matrix. The studies indicate that the microcapsule shell wall material does not play any significant role in defining the mechanical properties of the host composites. Yuan *et al*. [42] prepared a series of poly urea–formaldehyde (PUF) microcapsules containing epoxy resins and investigated the effect of surfactant type, surfactant concentration, adjusting time for pH value and heating rate on the size and surface morphology of microcapsules. No healing behavior of host matrix by the microcapsules prepared was reported in this study. Indeed, the UF capsules containing epoxy resins have been studied mostly for self-healing of polymer-based matrix by now and few researches have been reported on its utilization in concrete. Nishiwaki [43] prepared the UF formalin microcapsules filled with epoxy resin (the shell with the diameter of 20–70 µm) and the gelatin microcapsules with acrylic resin as a hardener (the shell with the diameter of 125–297 µm). The researcher suggested one component healing agent as the core material, the bigger microcapsules and the improved bond strength between the shell materials and cementitious matrix for use according to the experimental results. Instead, microencapsulation of UF shell and other core materials, such as sodium silicate, water, *etc*., has been more made for self-healing of concrete based on distinguished healing mechanisms, *i.e*., hydration or recrystallization due to chemical reaction between minerals in concrete and encapsulated healants [44,45,46]. However, this is a time-dependent healing process and thus it is difficult for strength restoration in a short period as compared to polymers used as the healing agent.

Though a number of studies have revealed various factors affecting the UF microcapsules containing epoxy resins, they are mainly aimed at its utilization in polymer-based composites rather than cementitious materials. Moreover, the material utilized must be appropriate to assist the microcapsules in enduring its required environment [47,48]. In the case of the application of self-healing concrete, the shell must be easily ruptured by cracks and rigid and strong enough to resist unexpected external forces during mixing of concrete. However, few researches have been reported on the mechanical properties of the shell. Taking into account these, the overall selection for the microcapsule process that depends on multiple factors as the mechanical properties, size distribution, and chemical nature, *etc.*, becomes the first focus of the present study. Furthermore, the self-healing efficiency of cement matrices by the microcapsules prepared was thereafter evaluated in terms of strength restoration and healing microstructure. 

## 2. Experiments

### 2.1. Materials

Melamine, urea, triethylamine, sodium dodecylbenzenesulfonate (SDBS) and formaldehyde (reagent mass fraction of 37%) were of analytical grade and supplied by Sinopharm Chemical Reagent Co., Ltd., China. N-butyl ether, hydrochloric acid (mass fraction 10%) and epoxy resin E-51 were commercially available from Hanzhong Materials Co., Ltd., Shanghai, China. Laboratory tap water was used. An epoxy-amine hardener from Shanghai Hanzhong Chemicals Co., Ltd., China, was used in the matrix for self-healing evaluation.

All paste specimens were made with ordinary Portland cement (OPC, Type I) and laboratory tap water. 

### 2.2. Preparation of Microcapsules

Melamine, urea and formaldehyde were dissolved under stirring in a three-necked flask at a molar ratio of 0.05:1:2. The pH value was subsequently adjusted to 8–9 using triethylamine. Then, the reaction system was heated up to 70 °C, and the mixture was stirred at reflux for 1 h to obtain a colorless transparent viscous melamine-urea-formaldehyde co-condensation resin prepolymer. Next, sodium dodecyl benzene sulfonate surfactant at a mass fraction of 0.5%~1% was added to the mixture, stirred for several minutes and cooled to room temperature. Then, E-51 epoxy resin containing a reactive diluent of n-butyl glycidyl ether (at a mass fraction of 17.5%) was added under stirring emulsification for 20–30 min. To form microcapsules, hydrochloric acid solution was slowly added to adjust the pH value to 2.0~4.0, and the temperature was increased to 60 °C. The solution was allowed to react for 2~3 h. Finally, the mixture was filtered, washed and dried to obtain a dry microcapsule powder. The microcapsule surfaces were moist, but no visible and dissociative water were observable in the particle system.

**Figure 1:**
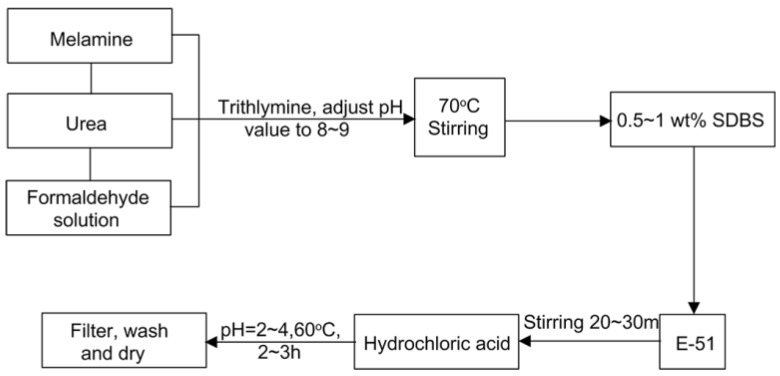
Synthesis procedure of microcapsules.

The synthesis procedure of the microcapsule powder is summarized in Figure 1. In general, the cost of the microcapsules in dry form is about 1.5$/100 g at present.

Cement paste with 0.3 w/c was used as the matrix. The epoxy-amine hardener, in an epoxy-to-amine ratio of 1.3, was mixed with the paste incorporating the microcapsules. The amine hardener was not added in the control samples. 1%, 2% and 4% of microcapsules by cement mass were added. The mixture was molded into 40 mm × 40 mm × 160 mm and cured under standard conditions for 28 d (20 °C ± 2 °C, ≥95% RH).

### 2.3. Test Procedure

A variety of synthesis conditions of the reaction system, including the core-wall mass ratio, the reaction temperature, pH value and stirring rate, were examined as given in Table 1. Only one condition changed, e.g., various core-wall ratio was prepared, while the remaining parameters were kept at the optimized value simultaneously, to reveal the effect of each specific condition on the prepared microcapsules.

#### 2.3.1. Morphology

Microcapsule emulsions were drop-wise added onto glass slides for optical observation of the morphology. Surface texture was also observed under SEM.

#### 2.3.2. Shell Thickness Measurement

Microcapsule shell thicknesses were measured using scanning electron microscopy. 

#### 2.3.3. Size Distribution

The size distribution of the microcapsule was examined using a Laser Particle Size Analyzer (Beckman Coulter Company, LS230). 

#### 2.3.4. Mechanical Properties of the Shell

An *in situ* nanomechanical testing system, TriboIndenter (Hysitron, USA), was used to measure the mechanical properties of the shell. 

In general, the samples were prepared under vacuum via curing, grinding and polishing following the below steps:

1. a certain amount of epoxy resin was placed in a mold;
2. dry microcapsule powder was mixed into the epoxy resin and stirred;
3. a layer of microcapsule powder was deposited at the bottom of another mold, and the epoxy resin-dry microcapsule mixture was poured over the deposited layer under vacuum.

**Figure 2:**
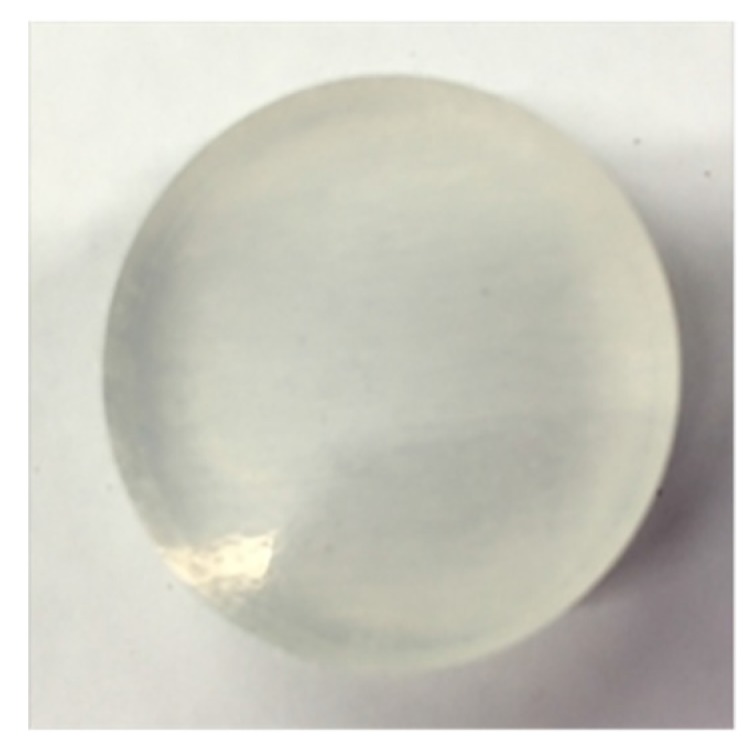
Sample for nanomechanical testing.

The samples, after 12 h left for curing of epoxy, were removed for grinding on Buehler-Met II paper disks with gradation of 15 μm, 13 μm, 11 μm, 8.5 μm and 6.5 μm, successively. For each gradation, the sample was ground for approximate 30 s under the pressure of 5N, followed by 30~60 min of burnishing with Buehler diamond suspensions in ethanol as polishing liquids of gradations 9 μm, 6 μm, and 3 μm until no obvious scratches were presented following the procedure of the previous step. The polishing was performed on a Buehler Consumables Texmat cloth and 30 min was allowed for each gradation. The samples were then placed in an ultrasonic bath for 10 min to clean the dust and unexpected particles. The surface quality was finally double checked and confirmed by optical microscopy to ensure a flat and smooth surface for tests as shown in Figure 2. To obtain reliable results, at least five indentations were performed on each specimen at different points on the desired objective. The Oliver and Pharr method was applied for analyzing the experimental data [49].

**Figure 3:**
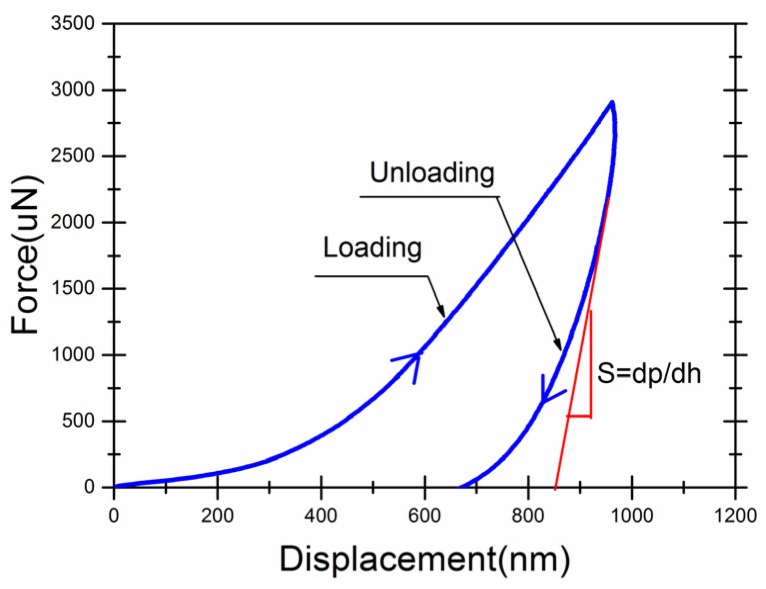
Typical load–displacement curve of nanoindentation and the indent image.

The nanoindentation tests were performed with a Hysitron Triboindenter fitted with a Berkovich tip (tip radius of 0.6 μm, angle of 142.3°). The indenter came into contact with the sample surface with a calibrated trapezoidal load function, as defined by a loading time of 5 s, a holding time of 2 s at a maximum load of 30 µN, and an unloading time of 5 s. A typical load–depth curve for indentation experiments was given in Figure 3. The contact stiffness (S) is defined as the slope of the unloading curve and given by [49,50,51]:

(1) $$S=\frac{dp}{dh}=\frac{2}{\pi }E_{r}\sqrt{A}$$

where A is the projected contact area at the peak load $P_{\max}$ and can be extrapolated by the Oliver and Pharr method [49], and $E_{r}$ is the reduced elastic modulus.

The hardness H is defined as

(2) $$H=\frac{P_{\max}}{A}$$

**Figure 4:**
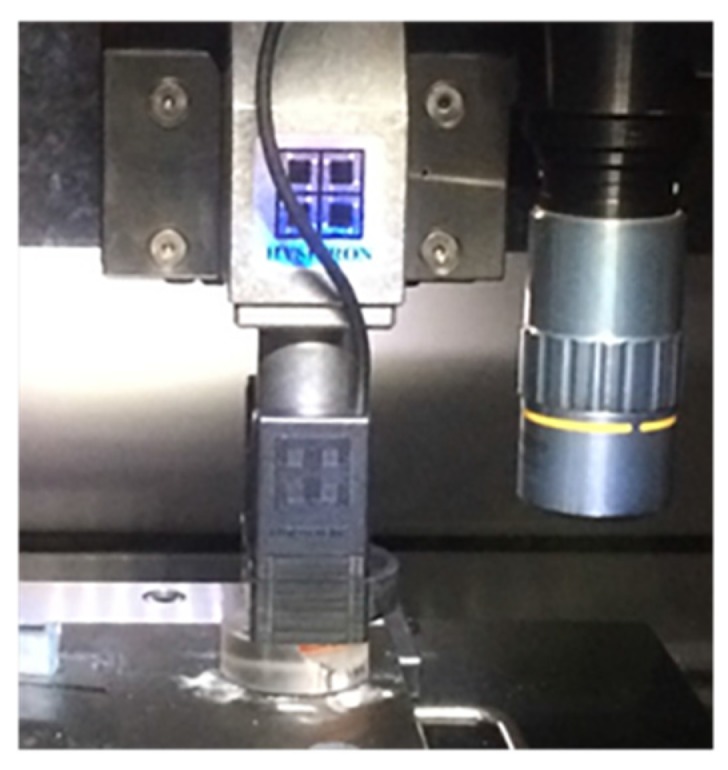
Testing setup.

It is noteworthy that the elastic moduli and/or hardness measured are supposed to be influenced by the epoxy matrix used to embed the sample due to a high gradient deformation between the capsules and the epoxy matrix. To diminish this unexpected effect, the epoxy with a elasticity comparatively close to that of the wall material of the microcapsules was selected to ensure the compatability in mechanical response. Figure 4 shows the test set up of TriboIndenter (Hysitron, USA).

#### 2.3.5. Chemical Structure of the Shell

Fourier transform infrared spectroscopy was used to identify the chemical structures of the microcapsules.

#### 2.3.6. Strength Restoration of Self-Healing Specimens

**Figure 5:**
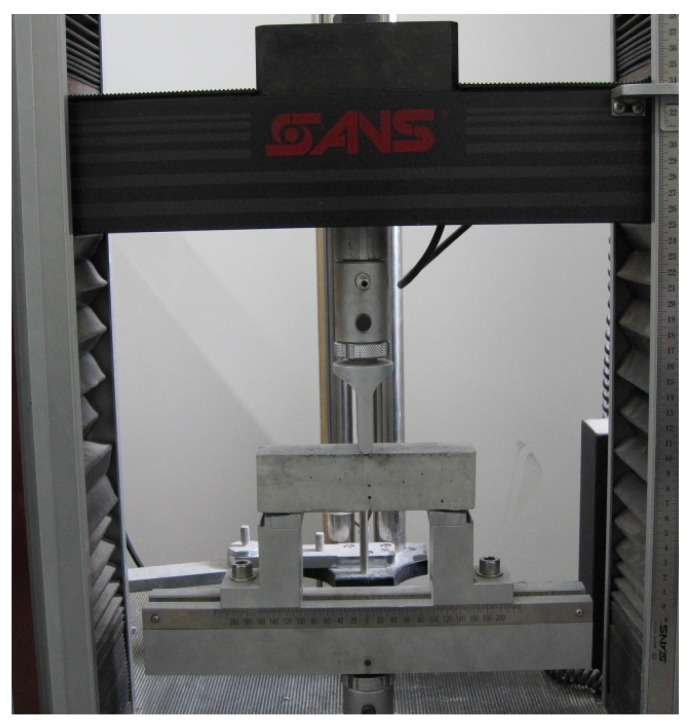
Three-point flexural loading setup.

Three-point flexural test was performed to evaluate the healing efficiency as shown in Figure 5. First, at least three paste specimens for each content of microcapsules were loaded to determine its maximum load resistance (denoted by f_max_). Then, the subsequent groups were loaded until 30%f_max_, 60%f_max_ and 80%f_max_, respectively. The pre-damage samples were left for 2 h for potential healing to occur. Then, the samples were reloaded until failure to determine the restored strength. Three replicates for each group were used to obtain the averaged results.

The flexural strength restoration rate of a sample is determined by

(3) $$\eta_{f}=\frac{R_{f}}{R_{f0}}$$

where $\eta_{f}$ is the flexural strength restoration rate (±0.01); $R_{f}$ the ultimate flexural strength (MPa) in reloading (±0.1 MPa); and $R_{f0}$ the initial flexural strength (MPa) (±0.1 MPa).

## 3. Results and Discussion

### 3.1. Morphology

**Figure 6:**
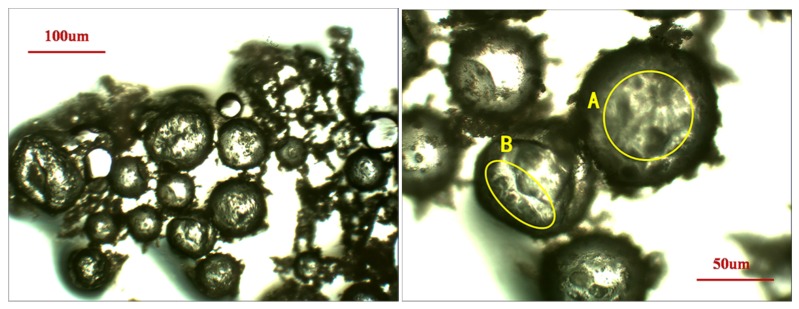
Optical microscopy images of microcapsules prepared.

The microcapsule morphology were observed using an optical microscope as shown in Figure 6. The sizes ranged from 10 µm–100 µm. The microcapsules were of regular spherical shapes, and the core and the shell were distinguishable. The surfaces were rough and were patterned with urea-formaldehyde resin nanoparticle deposits (marked by “A” in Figure 6). The deposited particles made the surface coarser, which can increase the contact surface area between the microcapsules and the cementitious matrix. Observed cracked shells (marked by “B” in Figure 6) may have been ruptured during the sample preparation.

**Figure 7:**
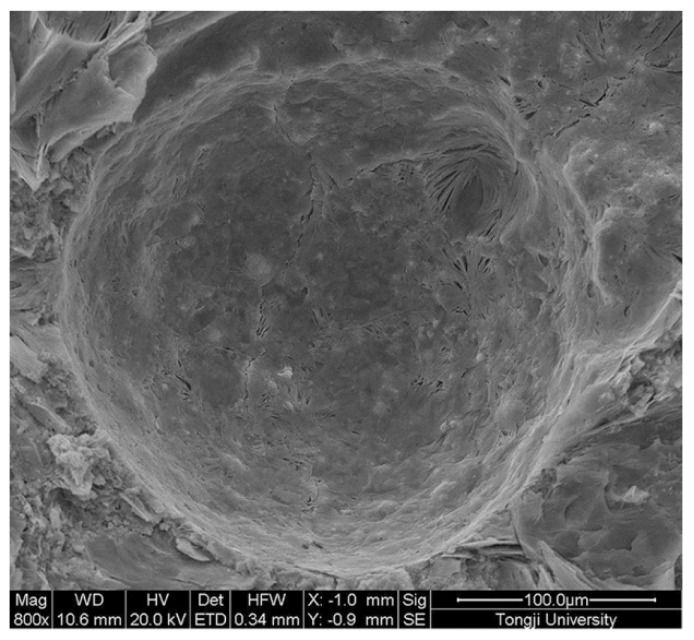
SEM observation of microcapsules morphology.

Figure 7 shows the surface texture of the microcapsules. The capsule is approximately 200 µm in diameter, and its surface looks rough with some small voids.This is beneficial to a good bond between the capsules wall and cementitious matrix. 

### 3.2. Particle Size and Distribution

**Figure 8:**
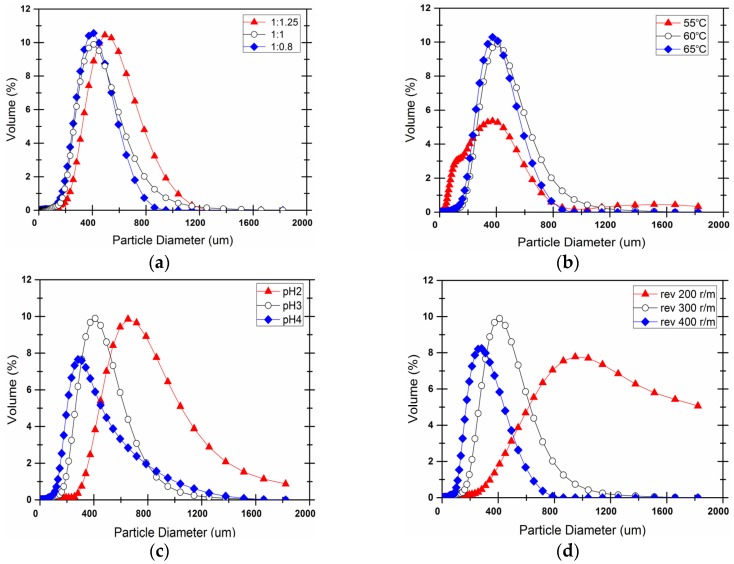
Effect of (**a**) core-wall ratio; (**b**) reaction temperature; (**c**) pH value and (**d**) stirring rate on the microcapsule size distribution.

Figure 8a shows the effect of core-wall ratio on the size distribution. The size shifts to the larger ones as the proportion of wall material increases, particularly for the core-wall ratio of 1:1.25. The excessive amount of shell material may lead to a more rapid deposition of resin particles on the surface and in turn, lead to a large increase in the size of the microcapsule. There is no signicant difference of the size distribution between the conditions of1:1 and 1:0.8 of core-wall ratio. One potential explanation for this is the irregular particles with too thin shells to encapsulate the increasing core material.

Figure 8b shows the effects of temperature on the size distribution. The microcapsules become in a wider spread range of size at 55 °C when compared with that of the other two temperatures. The volume at the peak size, *i.e*., about 400 µm, decreases half at 55 °C while it follows the quite similar distribution for 60 °C and 65 °C. At lower temperature, the reaction is not competitive and therefore the UF resin cross-linking reactions are slowand so is the particle formation rate. This results in a reduced ability of the shell to encapsulate the core materials. The size shifted slightly to the smaller ones when the temperature increased to 65 °C from 60 °C because the particles formed rapidly at a higher temperature. Under these conditions, the capsule shell is comparatively thin. The relatively large size of the capsule can be prone to rupture under the high shearing forces caused by stirring. However, the probability of collisions between the particles increases at high temperatures and this prevents the particles from depositing and adhering onto the core particles. 

Figure 8c shows the results for various pH values. As the pH increased, the chemical structure of the microcapsule shell became more compact and the shell was firmer. When the pH was three, the microcapsule size distribution was relatively uniform and larger, which contributed to the dense and uniform pre-formed shell resin particles. When the pH increased up to four, the shell resin particles had an uneven size distribution. Furthermore, the capsule particles were smaller because the UF resin could not condense. Fewer ether bonds formed in the shell, and the ability of shell to encapsulate core materials decreased. In general, as the pH decreased, the system reacted more rapidly. The raw material reacted at a higher rate, which results in a dense microcapsule shell and microcapsule with larger size.

If the reaction system and the physical parameters remained unchanged, the pH value and stirring rate are the main factors that affect the average particle size, as shown in Figure 8c,d. The diameter of the microcapsules reduced as the stirring rate increased. At higher stirring rates, the shear forces acting on the particle emulsion increased and the droplet size decreased, resulting in a relatively small microcapsule particle size. When the stirring rate decreased to 200 r/min, an abnormal increase in the average particle size of the capsule was observed. This may be attributed a too low stirring rate to provide enough shear force and the amount of kinetic energy in the system reduced accordingly, resulting in low microcapsule synthesis efficiency and encapsulation efficiency. Under these conditions, free epoxy-amine resins may coat the microcapsules and form large particles.

It should be noted that the core-wall ratio and the temperature do not show a significant effect on the size distribution unless they are changed to a either quite high or low value as compared to the other two factors of pH and stirring rate which result in a mononeous tendency of the size with increasing or decreasing of the two paramters. 

### 3.3. Mechanical Properties of Microcapsules 

The mechanical properties of the microcapsule wall directly affect the self-healing performance. When cracks form in the internal structure of cementitious materials, microcapsules at the tip of the crack are subjected to high stress force that can result in the rupture of the microcapsule wall. The healing agent encapsulated within the microcapsules is then released to fill the cracks. Therefore, the mechanical properties of the wall should be within an appropriate range for the potential breakage of the shell to occur. Other than this, the characteristics of the damage and/or cracks also play an important role in cracks healing. At a low mechanical load, crack defects develop slowly and thus the possibility for microcapsules to be ruptured decreases. Otherwise, crack defects propagate and increase in size at higher external load, which may lead to a large portion of unhealing cracks due to limited available healing agent in the system. Therefore, the ultimate healing efficiency is a critical combination of the mechanical properties of the microcapsule wall and the damage induced.

**Figure 9:**
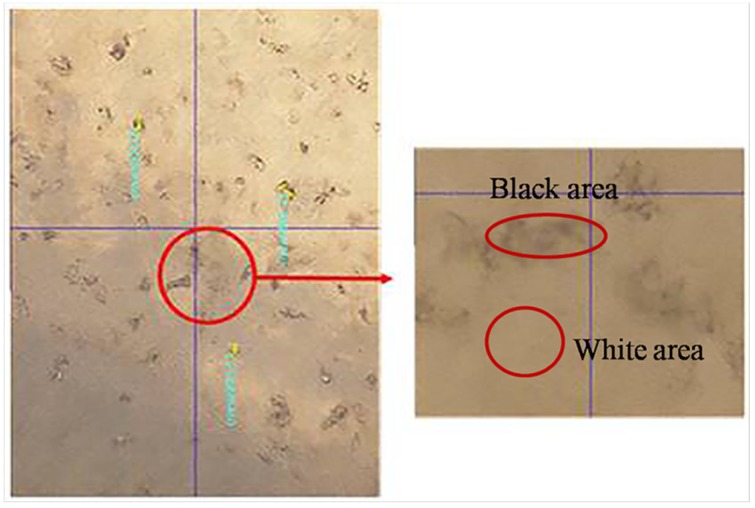
Inlays in the epoxy resin microcapsule reunion.

To find the appropriate testing zone for the properties of the microcapsules, a pure epoxy resin sample was prepared as calibration. The white and black areas of the microcapsules embedded sample were both tested as shown in Figure 9. 

**Figure 10:**
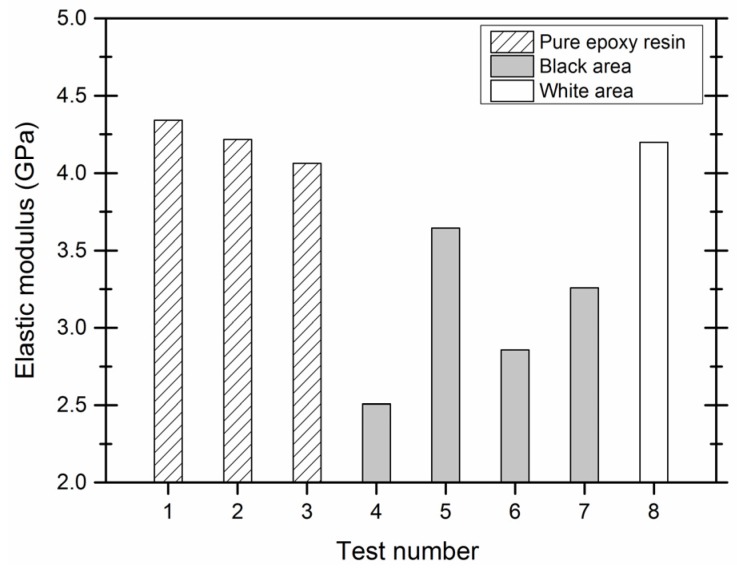
Elastic moduli of different areas in each specimen.

The elastic moduli of different areas in each specimen are shown in Figure 10. It can be seen that points 4~7 and 8 identify the black and white area of the microcapsule sample, respectively. The elastic moduli of the samples at points 1, 2, 3 and 8 were approximately 4 GPa. The elastic moduli of the samples at points 4, 5, 6 and 7 are distinguishable. These results indicated that the black area of the microcapsule samples is supposed to be the wall of microcapsules although it varies in a wide range.

**Figure 11:**
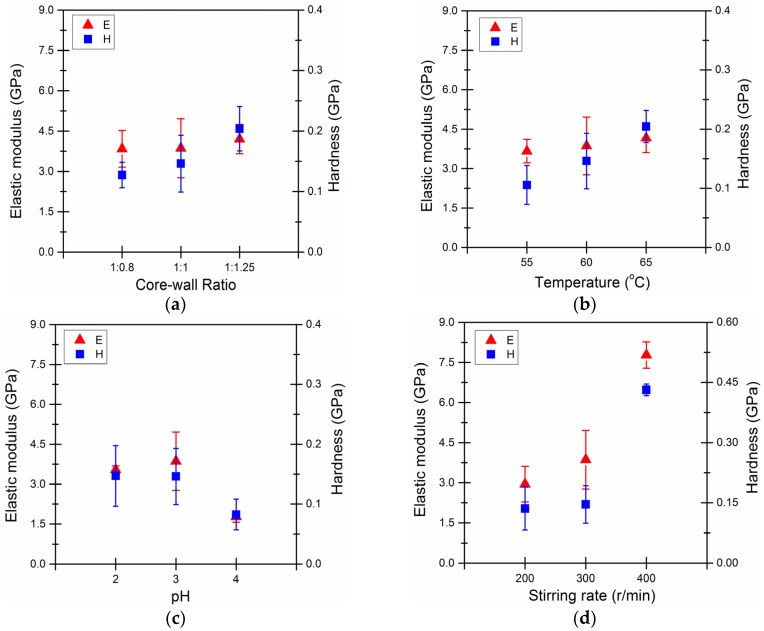
Effect of (**a**) core-wall ratio; (**b**) reaction temperature; (**c**) pH value and (**d**) stirring rate on elastic modulus (‘E’) and hardness of microcapsules (“H”).

Figure 11a shows the higher amount of wall materials used lead to the greater elasticity modulus as well as hardness. Under these conditions, the resin formed at a rapid rate and a thicker wall was generated. When the proportion of the core material is greater than the amount of the wall materials are supposed to encapsulate, the capsule wall elastic modulus and hardness values were lower. This was likely due to a lack of wall material, resulting in a thin capsule wall. Additionally, any unreacted epoxy may have been retained in the system and deposited as a coating on the microcapsule particle surface, which would also contribute to the lower elastic modulus and hardness.

Figure 11b shows that the microcapsule elastic modulus and hardness significantly increased at higher reaction temperatures. However, these values somewhat decreased when the reaction temperature was low. At low reaction temperatures, the urea-formaldehyde resin is unable to sufficiently react and the degree of resin cross-linking is low, thereby forming a thin wall. The bigger size of the microcapsules also accounts for the lower elastic modulus and hardness as represented in Figure 8b.

In Figure 11c, the three-dimensional network of chemical structure of the microcapsule wall was more compact and the wall was firmer for lower pH values. When the pH was 2~3, the elastic modulus and hardness were significantly greater than those at pH = 4. Depending on the polymerization mechanism and conditions of the formation of the wall molecules, the reaction rate and the degree of reaction are supposed to increase with the decreasing pH. Overall, these conditions resulted in a denser wall and higher mechanical indexes accordingly.

In Figure 11d, when the agitation rate increased to 400 r/min, the microcapsule elastic modulus and hardness significantly increased. This is likely due to the increased kinetic energy supplied to the capsules, resulting in the formation of denser walls. When the stirring rate decreased to 200 r/min, the available shear force and kinetic energy were too low. This resulted in low microcapsule synthesis efficiency, capsule core encapsulation efficiency and free epoxy resin coating. Therefore, these conditions resulted in a lower elastic modulus and hardness.

### 3.4. Chemical Structure of the Capsule Wall

The number of functional groups on the surfaces of the microcapsule walls was obtained by first analyzing the chemical structure of the microcapsule wall using infrared spectroscopy and subsequently determining the degree of reaction at the microcapsule wall. The system acidity is the primary factor that affects the chemical structure of the microcapsule wall and the reaction mechanism was analyzed by changes in pH.

A brief literature review [52] indicates the main characteristic infrared spectra peaks of epoxy resin E-51 are 771 cm^−1^, 1035 cm^−1^, 1184 cm^−1^, 1361 cm^−1^ and 1583 cm^−1^–774 cm^−1^. In the reaction of formaldehyde and urea, the number of hydroxyl and amino groups gradually decreased, and the absorption peaks of 3330 cm^−1^ and 1000 cm^−1^ decreased.

**Figure 12:**
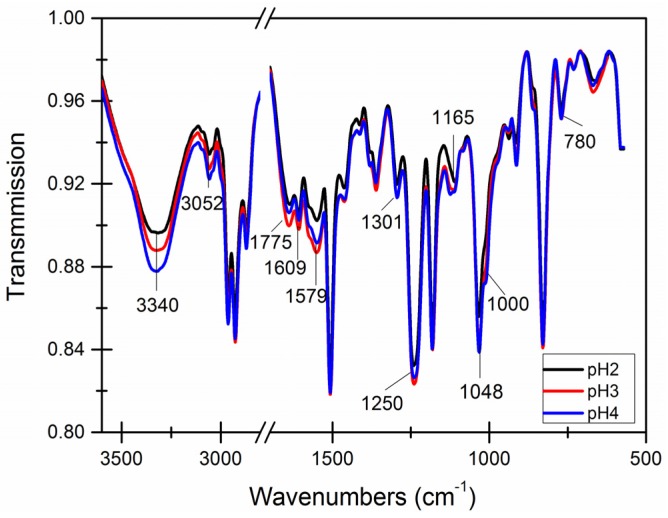
Infrared spectroscopy of microcapsules with different pH values.

Figure 12 shows decreases in the O−H bond peak (3340 cm^−1^) as the pH decreased; this may indicate that low pH aids in the formation of R−O−R′ bonds. Decreases in the N-H bond peak (1000 cm^−1^) were more obvious and was only observed when the pH was 4. A large number of R−O−R′ bonds (1250 cm^−1^ and 1084 cm^−1^) formed, which can further react to the form the wall. 

Dramatic decreases in epoxy resin prominent peaks (780 cm^−1^, 1165 cm^−1^, 1301 cm^−1^ and 1579 cm^−1^–1175 cm^−1^) were observed, indicating a higher amount of epoxy was encapsulated in the microcapsules. The prominent peaks of epoxy resin was more obvious when the pH was 4.

**Figure 13:**
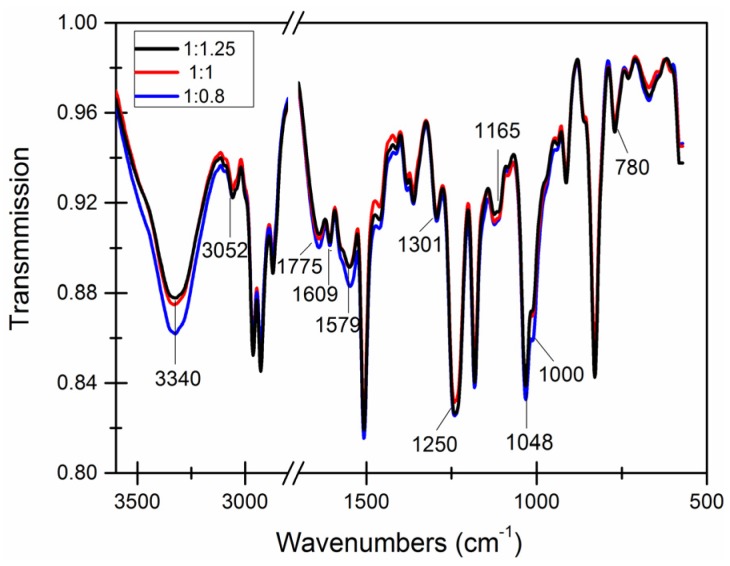
Infrared spectroscopy of microcapsules with different core-wall ratios.

Figure 13 shows that the absorption peak of residual hydroxyl groups (3340 cm^−1^) decreased as the increasing amount of wall material. Weak absorption peaks of amido groups (1000 cm^−1^) were observed only when the content of the core material was larger than the content of the wall material. The numbers of hydroxyl and amido groups in the formaldehyde and urea reaction gradually decreased as the amount of wall material increased. As the amount of wall material increased, epoxy resins were sealed within the denser package, which stabilizing the chemical structure of capsule surface. The number of characteristic peaks of epoxy resin at 780 cm^−1^, 1165 cm^−1^, 1301 cm^−1^ and 1579 cm^−1^–1775 cm^−1^ also decreased. This indicated an increased encapsulation efficiency.

## 4. Self-Healing Efficiency of Cement-Based Material by Microcapsules Prepared

**Figure 14:**
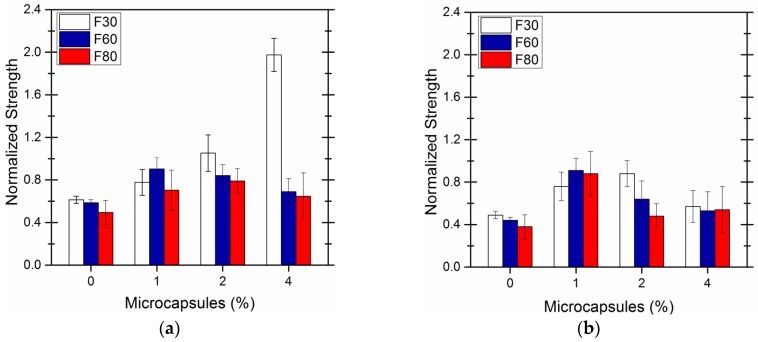
Flexural strength restoration rate of specimen with different content of microcapsules prepared: (**a**) in optimized parameters; (**b**) with a core-wall ratio of 1:1.25.

The microcapsules were prepared using the optimized parameters mentioned above as shown in Figure 14a. In Figure 14b, microcapsules were prepared with a core-wall ratio of 1:1.25 for comparision.

The specimens incorporating the microcapsules prepared using optimized parameters show more obvious healing efficiency in terms of strength restoration. The maximum flexural strength restoration was seen at approximately 1.8 for 4 wt % of microcapsules and pre-damage of 30%f_max_. The strength restoration becomes much less for 1 wt % and 2% wt of microcapsules as 0.8 and 1.0, respectively when the specimens were preloaded by 30%f_max_. 

Concerning the effects of pre-damage on the strength restoration rate, the flexural strength of the specimens for different microcapsule contents were generally high at 30%f_max_. This is due to that the cracks or damages are large in size, which results in incomplete healing of cracks by the adhesive that is limited avalible in the system. Under pre-damages of 60%f_max_ and 80%f_max_, the strength restoration rate was slightly higher at 1 wt % or 2 wt % microcapsule content. The flexural strength restoration rate decreased with the degree of pre-damage for the blank specimens. This indicates that the increased damage compromised the remaining load resistance of the specimens. 

The strength restoration of the sample with 1 wt % of microcapsules was more obvious than that of 2 wt % and 4 wt %. Concerning the effect of pre-damage on the strength restoration rate, 30%f_max_ led to the best strength recovery for the microcapsules prepared in optimized parameters since it passed a critical combination of a high enough stress to active cracks healing but not too high to induce extra cracks that cannot be healed by the limited available adhesive. The one with a core-wall ratio of 1:1.25 does not show a clear trend of the strength recovery with an increase of load applied.

**Figure 15:**
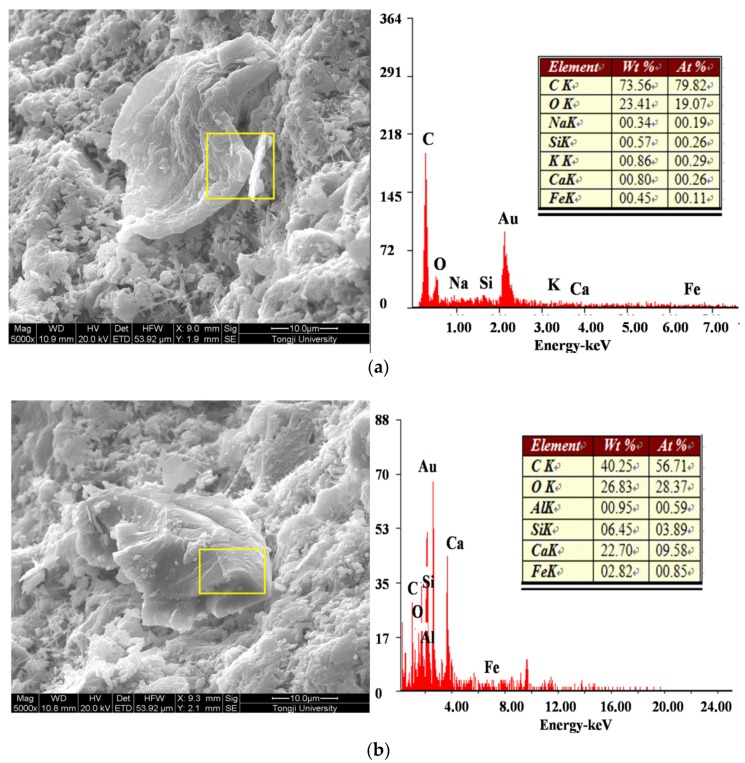
Residual microcapsule shell after outflow of healing agent: (**a**) core-wall ratio 1:1; (**b**) core-wall ratio 1:1.25.

**Figure 16:**
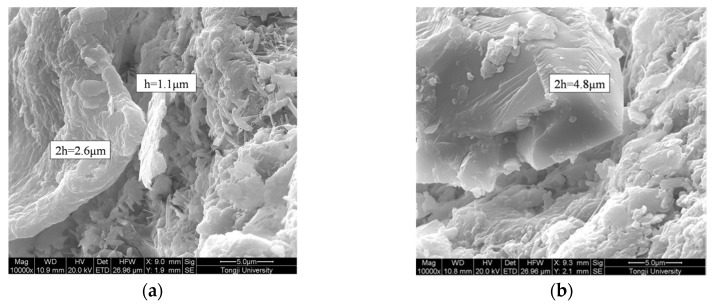
Measurement of microcapsule shell by SEM: (**a**) core-wall ratio 1:1; (**b**) core-wall ratio 1:1.25.

Figure 15a,b show a residual microcapsule shell of a ruptured microcapsule with 1:1 and 1:1.25 of the core-wall ratio, respectively. The shell thicknesses of the two types of microcapsules were measured using SEM as shown in Figure 16. When the core-wallratio was 1:1, the shell thickness ranged from 1.1~1.3 μm. As the content of the shell material increased, resin particle more rapidly formed, which depositing more microcapsule particles on the surface. In this way, the size of the microcapsule and the shell thickness increased to 2.4 μm. The shell thickness of the microcapsule has a significant effect on the stability and cohesiveness, particularly on the mechanical properties of the microcapsules. If the microcapsule shell is too thin, the capsules can be easily damaged during the storing process or mixing of the paste specimens, which results in the premature release of healing agent. However, if the shell is too thick, the microcapsules are unable to be ruptured by internal stresses in the cement paste to achieve cracks healing. For example, for microcapsules prepared with a core-wall ratio of 1:1.25, the excessive amount of shell material resulted in a more rapid deposition of resin particles on the shell surface. This in turn resulted in increases in the size of the microcapsule and the shell thickness. As a result, the microcapsule could not be ruptured by internal stresses, which may lead to failure of cracks healing.

## 5. Conclusions

The microcapsules for self-healing of cementitious materials were prepared by an *in situ* method using malmine urea-formaldehyde as a shell and epoxy resin as an adhesive. The properties of the microcapsules prepared in a variety of synthesis conditions were characterizede. The healing efficiency of cement paste by the microcapsules prepared was evaluated in terms of the strength restoration as well as the healing microstructure A few conclusions can be drawn as follow: 

The four factores, reffering to stirring rate, pH value, core-wall ratio, reaction temperature, show some influences on the microcapsules prepared. For the size distribution, the stirring rate and pH value exhibited more significant effect as compared to the other two factors. For the mechanical property of the shell, the elasticity modulus and hardness of shell improved with an increase of the proportion of shell material used due to an improved encapsulation ability of the melamine urea-formaldehyde resin . When the pH was relatively low, the three-dimensional structure was more solid and the chemical structure was more stable the elastic modulus of the shell increased accordingly. An increase in temperature was attributed to a higher degree of shell condensation polymerization and therefore the enhanced mechanical properties. Higher stirring rates increased the amount of kinetic energy available and benefited the formation of the microcapsules, which resulted in an increase of mechanical indexes. Overall, these factors suggest an optimal synthesis scheme: mixing a core-wall ratio of 1:1 at an optimum reaction temperature of 60 °C while maintaining the pH at 2~3 at a stirring rate of 300–400 r/min.

The healing strength of damaged cement samples containing the microcapsules prepared under optimized conditions was much more obvious than the microcapsules prepared with a core-wall ratio of 1:1.25. The strength restoration rate is closely related to the capsule dosage, level of pre-damage and type of microcapsule used. For a common level of damage, the strength restoration rate at a 1 wt % microcapsule dosage was the most. While for the effect of damage degree, the samples subjected to 30% f_max_ pre-damage showed the best strength restoration rates. In conclusion, the final healing efficiency depends on the combined effect of a high enough stress to active cracks healing but not too high to induce extra cracks in big size that cannot be healed by the adhesive that is limited available in the system.

## Figures and Tables

**Table 1:** Microcapsule preparation parameter.

Core-Wall Ratio	Reaction Temperature (°C)	pH Value	Stirring (r/min)
1:0.8	60	3	300
1:1	60	3	300
1:1.25	60	3	300
1:1	55	3	300
1:1	60	3	300
1:1	65	3	300
1:1	60	2	300
1:1	60	3	300
1:1	60	4	300
1:1	60	3	200
1:1	60	3	300
1:1	60	3	400
